# Supplementary material for: The amyloid packing difference: A pairwise comparison metric for amyloid structures
Source: Structure. Author manuscript; Available in PMC 2026 Aug 10. (PMC7619344; doi:10.1016/j.str.2026.04.014)
Supplement: Supplementary file 1 [file EMS217164-supplement-Figs_S1___S4.pdf]

Structure, Volume *34*

Supplemental Information

The amyloid packing difference: A pairwise comparison metric for amyloid structures

Sjors H.W. Scheres

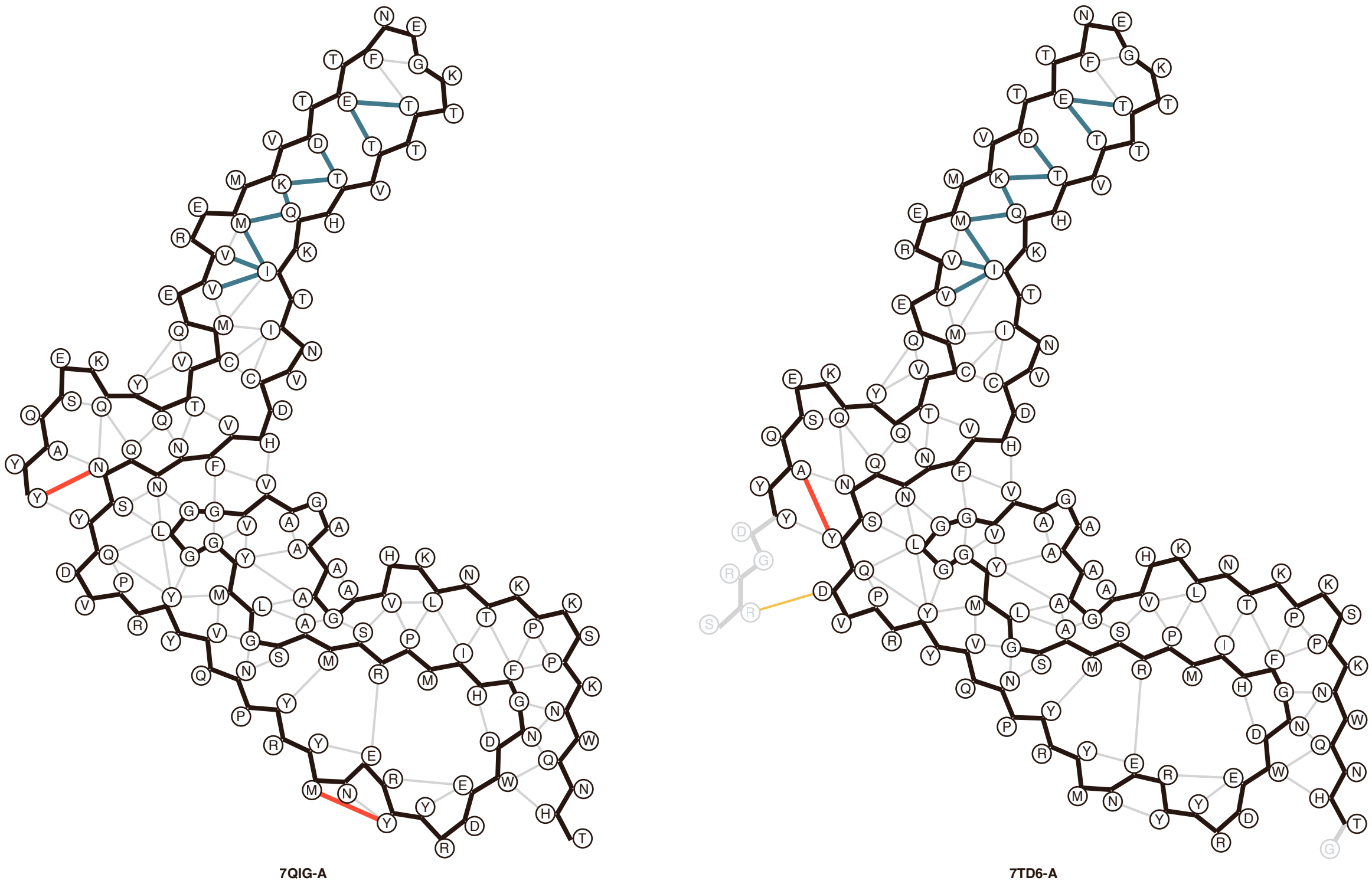

Legend

- common residue
- extra residue
- mutated residue
- right-left flip
- backbone (CA-CA)
- unique intra contact
- unique inter contact
- extra contact
- common contact (Z-offset=0)
- common contact (Z-offset=1)
- common contact (Z-offset=2)
- common contact (Z-offset>=3)

Summary

Protofilament 0

- residues in structure 1:	132
- residues in structure 2:	138
- residues in common:	132
- max(extra residues):	6
For common residues:	
- right-left flips:	0
- uniq intra-contacts:	7
- Z-offsets:	11
- XY-differences:	7
- XYZ-differences:	18
--	
**- PF0 Amyloid Packing Difference (XY):**	**9%**
**- PF0 Amyloid Packing Difference (XYZ):**	**17%**

Figure S1: Output of the compare.py script for PDB entries 7QIG and 7TD6, related to Figure 1.

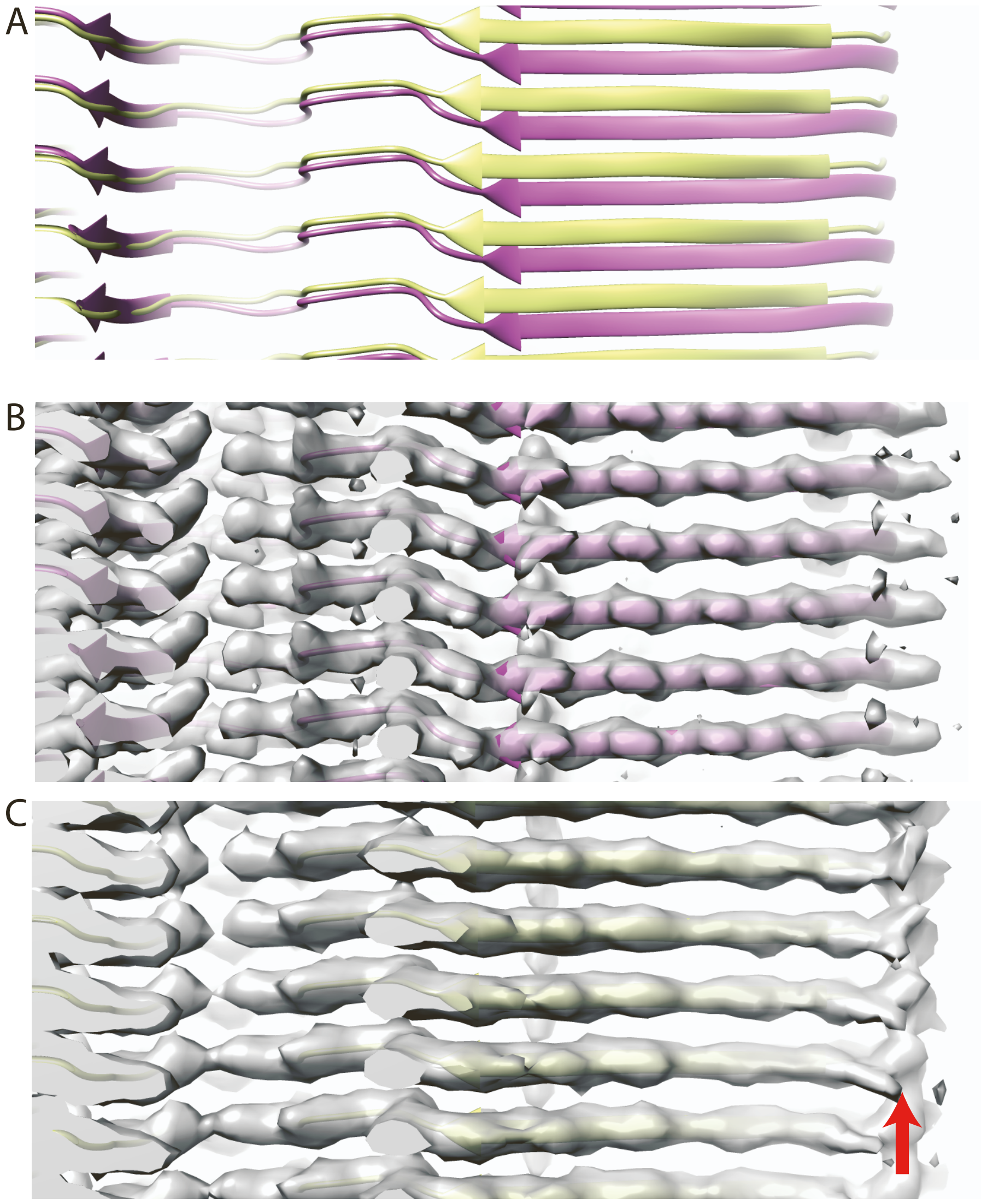

Figure S2: Different Z-tracings in PDB entries 7QIG and 7TD6, related to Figure 1. A. Superposed main-chain trace of PDB entries 7QIG (pink) and 7TD6 (yellow). **B.** Main-chain trace and cryo-EM density of structure 7QIG. **C.** As B, but for 7TD6. The red arrow indicates spurious connecting densities between layers of the amyloid that are indicative of the reconstruction being the result of a refinement getting stuck in a local minimum.

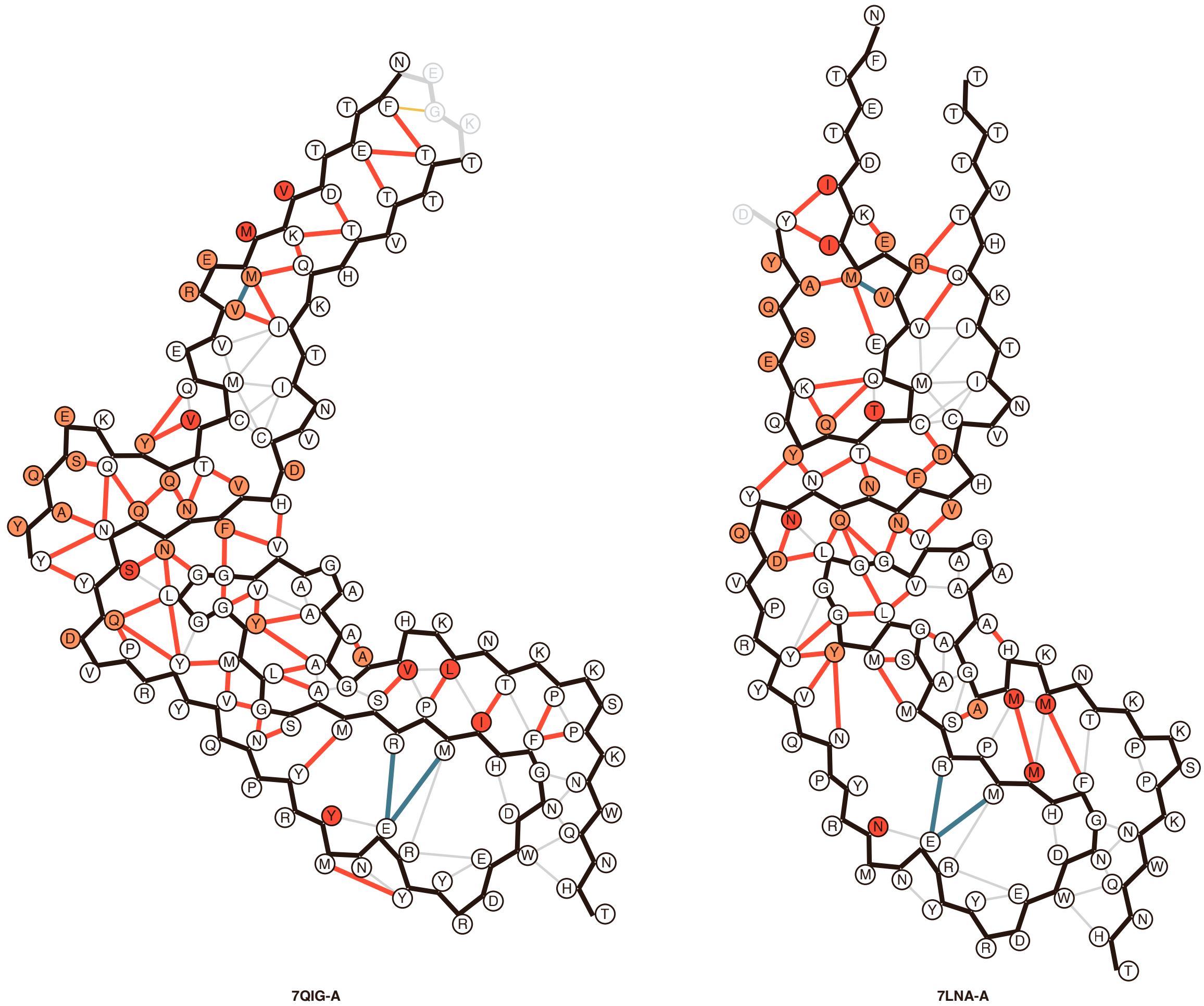

Legend

- common residue
- extra residue
- mutated residue
- right-left flip
- backbone (CA-CA)
- unique intra contact
- unique inter contact
- extra contact
- common contact (Z-offset=0)
- common contact (Z-offset=1)
- common contact (Z-offset=2)
- common contact (Z-offset>=3)

Summary

**Protofilament 0**	
- residues in structure 1:	132
- residues in structure 2:	130
- residues in common:	129
- max(extra residues):	3
For common residues:	
- right-left flips:	21
- uniq intra-contacts:	74
- Z-offsets:	5
- XY-differences:	77
- XYZ-differences:	80
**- PF0 Amyloid Packing Difference (XY):**	**61%**
**- PF0 Amyloid Packing Difference (XYZ):**	**63%**

Figure S3. Output of the compare.py script for PDB entries 7QIG and 7LNA, related to Figure 1.

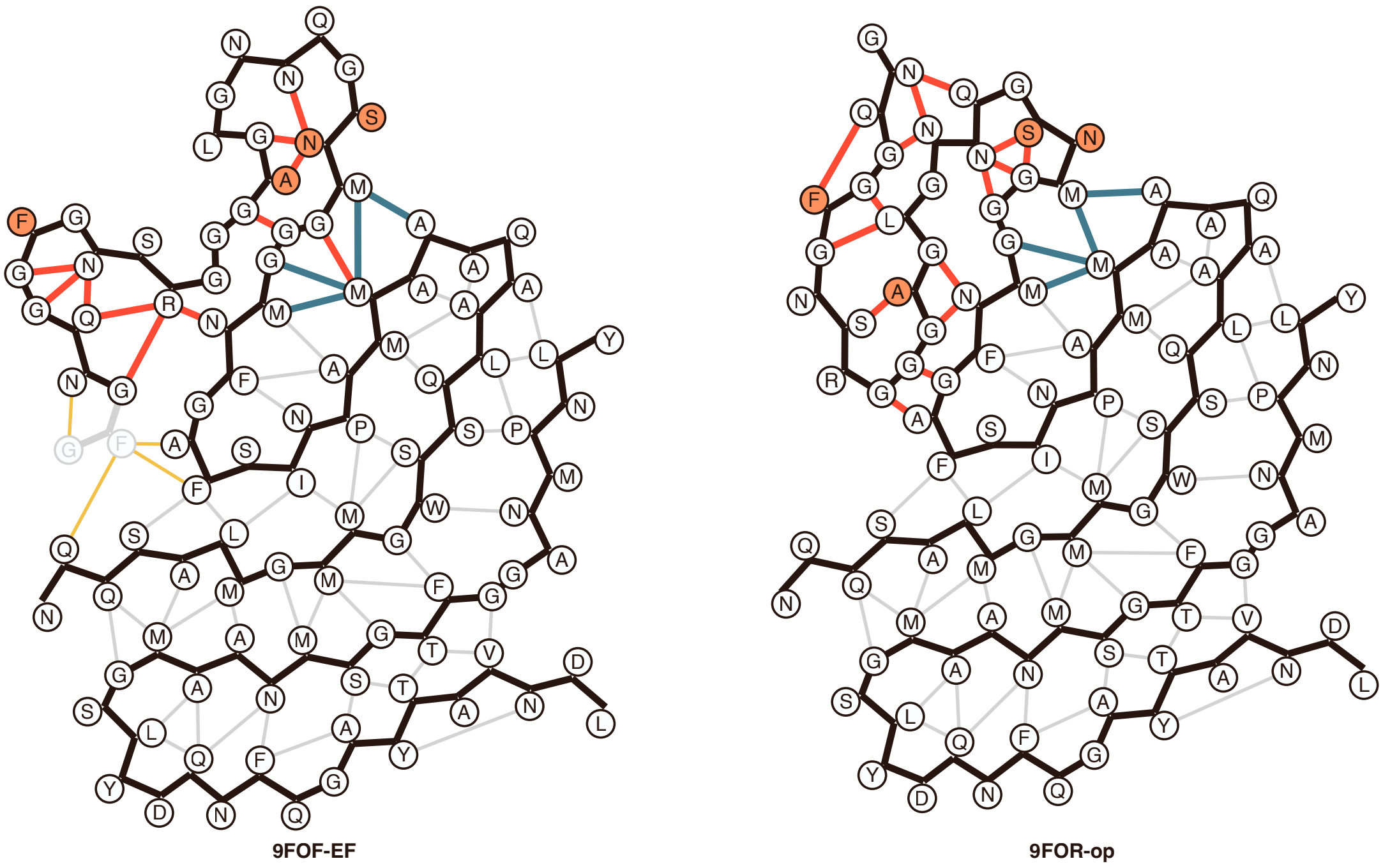

Legend

- common residue
- extra residue
- mutated residue
- right-left flip
- backbone (CA-CA)
- unique intra contact
- unique inter contact
- extra contact
- common contact (Z-offset=0)
- common contact (Z-offset=1)
- common contact (Z-offset=2)
- common contact (Z-offset>=3)

Summary

Protofilament 0

- residues in structure 1:	36
- residues in structure 2:	36
- residues in common:	36
- max(extra residues):	0
For common residues:	
- right-left flips:	0
- uniq intra-contacts:	0
- Z-offsets:	0
- XY-differences:	0
- XYZ-differences:	0
--	
**- PF0 Amyloid Packing Difference (XY):**	**0%**
**- PF0 Amyloid Packing Difference (XYZ):**	**0%**

Protofilament 1

- residues in structure 1:	64
- residues in structure 2:	62
- residues in common:	62
- max(extra residues):	2
For common residues:	
- right-left flips:	4
- uniq intra-contacts:	29
- Z-offsets:	5
- XY-differences:	29
- XYZ-differences:	33
--	
**- PF1 Amyloid Packing Difference (XY):**	**48%**
**- PF1 Amyloid Packing Difference (XYZ):**	**55%**

Protofilament interface 0-1

- residues in left:	19
- residues in right:	19
- residues in common:	19
- max(extra residues):	0
For common residues:	
- Z-offsets (0/19):	0.0%
- right-left flips (0/19):	0.0%
- uniq inter-contacts (0/19):	0.0%
--	
**- PF0-PF1 Amyloid Packing Difference (XY):**	**0.0%**
**- PF0-PF1 Amyloid Packing Difference (XYZ):**	**0.0%**

Figure S4: Output of the compare.py script for PDB entries 9FOF and 9FOR, related to Figure 3. The ADP calculated over both protofilaments in PDB entries 9FOF and 9FOR is (29+2) /(64+36), or 31 %.
